# Examining the Role of Resilience and Hope in Grit in Multiple Sclerosis

**DOI:** 10.3389/fneur.2022.875133

**Published:** 2022-05-16

**Authors:** Beatrice Lee, Phillip Rumrill, Timothy N. Tansey

**Affiliations:** ^1^Department of Counseling, Educational Psychology and Special Education, Michigan State University, East Lansing, MI, United States; ^2^Human Development Institute, University of Kentucky, Lexington, KY, United States; ^3^Department of Rehabilitation Psychology and Special Education, University of Wisconsin-Madison, Madison, WI, United States

**Keywords:** grit, positive psychiatry, multiple sclerosis (MS), resilience, hope, positive psychology

## Abstract

The purpose of this study was to examine the effects of resilience and hope on grit when controlling for demographic covariates, depression, and anxiety in people with multiple sclerosis (MS). This was a cross-sectional study with a sample of 348 participants with MS. Descriptive statistics were performed to examine participants' demographic characteristics. A three-step hierarchical regression analysis was conducted to evaluate the extent to which resilience and hope explain the unique variance in grit while controlling for demographic covariates, depression, and anxiety. Findings suggested that resilience and hope explained a significant amount of variance in grit when controlling for demographic covariates, depression, and anxiety. Furthermore, higher resilience and hope scores were associated with higher grit scores. Given that resilience, hope, and grit are modifiable, rehabilitation and mental health professionals (e.g., psychologists, psychiatrists, rehabilitation counselors) can integrate strength-based interventions into their practices to bolster resilience, hope, and grit in people with MS. Our paper also has implications for interdisciplinary research and clinical practice.

## Introduction

Multiple sclerosis (MS) is a chronic immune-mediated disease that affects nearly one million individuals in the United States (1, 2). People with MS experience unpredictable disease course, unpleasant symptoms, and psychological difficulties (3). Additionally, the early onset of MS (20 to 40 years of age) can cause considerable psychosocial disruptions to life goals, employment, and relationships (4, 5). Given that the MS disease process is highly variable from person to person, the experiences of living with MS and quality of life are individual and personal (6, 7). Individual's perception of the disease and one's ability to effectively cope is crucial in health maintenance and psychological wellbeing (8).

The intrusiveness and ambiguity associated with MS can further lead to developing psychological distressing symptomatology (9). Psychiatric symptoms, such as depression and anxiety, are common in persons with MS (4, 10). In a systematic review and meta-analysis, the prevalence of depression was reported to be 30.5 and 22.1% for anxiety in MS (10). Moreover, the explanations for comorbid depression and anxiety are multifactorial, including MS-related biological processes (e.g., changes in brain structure), emotional and cognitive reactions to the unpredictable nature of the disease, and psychosocial factors (10). For example, depression in MS can be influenced by various psychosocial factors, including feelings of helplessness, poor relationships, high levels of stress, and insufficient coping strategies (11). Additionally, anxiety in MS has been linked to low self-efficacy and higher levels of disability and stress (12). Depression and anxiety are disabling symptoms that can contribute to lower quality of life, decreased treatment adherence, increased symptom burden, and poorer health and functional status in persons with MS (4, 7, 10, 13, 14).

Shifting away from pathology and impairments, positive psychology emphasizes on building positive qualities that allow individuals to flourish (15, 16). A strength-based paradigm offers a more holistic understanding of the human experience and foster positive health and mental health (15, 17). Strengthening protective factors can buffer against psychopathology and promote positive mental health (18, 19), which is consistent with the positive psychology and strength-based framework of identifying and enhancing individual's assets. Furthermore, protective factors may reduce individuals' vulnerability of developing psychiatric problems because they have protective resources when encountering adversity (18).

Grit is conceptualized as perseverance and passion for long-term goals despite adversity (20). Grit comprises two factors, including perseverance of efforts and consistency of interests (20, 21). Perseverance of efforts is described as people's ability to continue their efforts even when facing setbacks, while consistency of interests refers to people's ability to sustain interests toward future goals over time (20). People who have higher levels of grit were more likely to have better achievements (e.g., educational attainment, fewer career changes) that are beyond talents (20). Furthermore, those with higher grit had a lower tendency to drop out from life commitments (22).

Grit has been suggested to be associated with enhanced health and mental health outcomes, and psychological wellbeing in people with disabilities and health conditions (23–26). For instance, in a study examining grit in college students with chronic health conditions, researchers found that grit is negatively associated with depressive and anxious symptoms, and is positively associated with wellbeing (25). In a study on adults with HIV demonstrated that higher grit was associated with functional independence (24). Similarly, another study found that veterans with mental illnesses with higher levels of grit were less likely to experience functional disability (26). In a study on people with Parkinson's disease, researchers found that individuals with higher levels of grit were more likely to use more positive coping strategies and indicate higher quality of life (23).

Protective factors, including resilience and hope, are also positive psychology constructs that have been demonstrated to promote health and mental health outcomes in people with disabilities (27–29). Resilience is one's ability to achieve growth and flourish in the face of adversity (30), whereas hope is the cognitive-motivational state when individuals have the cognitive energy to work toward their goals and their ability to generate paths to meet their goals (31). Despite facing various physical and psychological challenges, it is possible for people with MS to experience positive adjustment (32) and positively influence their psychological wellbeing. Resilience and hope were inversely associated with depression and anxiety in people with MS (33, 34). Resilience interventions have been demonstrated to improve mental health outcomes in MS (35, 36). In a similar fashion, a hope-based group therapy was demonstrated to reduce depression and anxiety symptoms in a community sample (37). This suggests that importance of identifying and building protective factors to enhance mental health in people with MS.

Some studies have examined the relationship among grit, resilience, and hope in individuals without disabilities. Specifically, grit and resilience have been studied to understand student success in academic settings (38, 39), whereas hope was identified as a strong predictor of grit in academic settings (40) and was discussed as a potential contributor to grit in a disadvantaged sample (41). For example, in a sample of healthy adults, grit moderated the effect of resilience on pain tolerance (42). Hope was found to be the strongest predictor of grit among Latina/o college students, suggesting the association between hope and grit (40). However, there are relatively limited research examining resilience and hope and their associations with grit in people with disabilities.

While other positive psychology constructs (e.g., resilience, hope, core self-evaluations, social support) have been studied in people with MS (33, 35, 43–46), there is a dearth of research examining grit in this population. To our knowledge, this is the first study to examine grit in people with MS. Additionally, given prior research has demonstrated the role of resilience and hope in relation to grit [e.g., (39, 40)], this study aimed to further examine these associations to contribute to the MS literature. Given the potential role of grit in buffering against psychopathology and enhancing health and rehabilitation outcomes, it is imperative to gain a better understanding of grit. These findings might inform the integration of hope, resilience, and grit interventions in clinical and research settings. Therefore, the purpose of the study was to explore the effects of resilience and hope on grit when controlling for demographic covariates, depression, and anxiety.

## Methods

### Procedures

Subsequent to the Institutional Review Board (IRB) approval by the University of Wisconsin-Madison, the study invitation with the online survey link was advertised through the National Multiple Sclerosis Society (NMSS) website and was sent via emails to NMSS members. The inclusion criteria for the study included being at least 18 years old and having a MS diagnosis. The survey included an informed consent statement and information about the study following the IRB requirements. Before filling out the survey, participants had to agree to participate in the study. After completing the survey, participants were directed to another link and had the option to provide their names and emails to receive a $10 gift card. As outlined in the consent form, the first 250 participants who participated in the study received the gift card. To ensure confidentiality, participants' names and emails were separated from their survey responses. While 446 participants initiated the survey, 73 participants provided incomplete data. Therefore, there were 373 participants who completed the full survey.

### Measures

#### Demographic and MS Characteristics

A demographic questionnaire was used to ask participants to indicate their demographic (e.g., age, self-reported sex, race, marital status, education level, employment status) and MS characteristics (e.g., MS subtype, MS duration).

#### Depression and Anxiety

Depression and anxiety were measured using the *Patient Health Questionnaire-4 (PHQ-4)* developed by Kroenke et al. (47). The *PHQ-4* includes two depression items (e.g., “*little interest or pleasure in doing things.”*) and two anxiety items (e.g., “*feeling nervous, anxious, or on edge.”*). Each item is rated on a 4-point Likert scale, ranging from 0 (not at all) to 3 (nearly every day). In the present study, the depression subscale (the sum of the two depression items) and anxiety subscale scores (the sum of the two anxiety items) were computed where each subscale's total score ranges from 0 to 6. Higher scores indicate higher levels of depressive and anxiety symptoms. Kroenke et al. (47) reported the internal consistency reliability coefficient to be α = 0.81 for the depression subscale, and α = 0.82 for the anxiety subscale. The Cronbach's alpha of the depression subscale and the anxiety subscale in the current study were computed to be α = 0.85 and α = 0.88, respectively.

#### Resilience

Resilience was measured utilizing the *Brief Resilience Scale (BRS)* developed by Smith et al. (48). The *BRS* comprises six items (e.g., “*I tend to bounce back quickly after hard times.”*) assessing one's ability to bounce back from stressful events with three positively worded items and three negatively worded items. Each item is rated on a 5-point Likert scale, ranging from 1 (strongly disagree) to 5 (strongly agree). Negative worded items are reverse scored. Higher scores represent higher levels of resilience. The item scores were summed to a total score and averaged. Smith et al. (48) reported the internal consistency reliability coefficients to range from 0.80 to 0.91. In the current study, the Cronbach's alpha was computed to be 0.88.

#### Hope

Hope was measured using the *Trait Hope Scale (THS)* developed by Snyder et al. (31). The *THS* comprises 12 items (e.g., “*I can think of many ways to get out of a jam.”*), including 4 items measuring pathways thinking, 4 items measuring agency thinking, and 4 filler items. Each item is rated on a 4-point Likert scale, ranging from 1 (definitely false) to 4 (definitely true). The item scores were summed to a total score and averaged. Higher scores indicate higher hope. Snyder et al. (31) reported the internal consistency reliability coefficients to range from 0.74 to 0.84. In the present study, the Cronbach's alpha was 0.89.

#### Grit

Grit was measured using the *Short Grit Scale (Grit-S)* developed by Duckworth and Quinn (21). The Grit-S comprises 8 items assessing one's consistency of interest and perseverance of effort (e.g., “*New ideas and projects sometimes distract me from previous ones.”*), including 4 positively worded items and 4 negatively worded items. Each item is on a 5-point Likert Scale, ranging from 1 (not like me at all) to 5 (very much like me). Negative worded items are reverse scored. The item scores were summed to a total score and averaged. Higher scores represent higher levels of grit. Duckworth and Quinn (21) reported the internal consistency reliability coefficients to range from 0.73 to 0.84. In the present study, the Cronbach's alpha was 0.77.

### Data Analysis

All analyses were conducted using the Statistical Package for the Social Sciences for Mac (SPSS version 25.0). For data quality check, a Cook's Distance outlier analysis was computed by using the formula of 4/(n-k-1). Using the general rule of thumb (49), Cook's distance was computed to be 4/(373-7-1) = 0.0109589041. Twenty-five cases were identified as outliers and were removed from regression analysis, resulting in a final sample size of 348 participants. Other assumption checks were also performed, which the plot suggested the linear relationship between independent variables and the dependent variable. The Durbin-Watson test showed a value of 2.02, which demonstrates independence of residuals. Reliability for each scale was computed and reported above. The variance inflation factors values ranged from 1.03 to 2.46, indicating no multicollinearity.

Descriptive statistics were performed to examine participants' demographic characteristics. A three-step hierarchical regression analysis was conducted to evaluate the extent to which resilience and hope explain the unique variance in grit while controlling for demographic covariates, depression, and anxiety. Demographic variables (i.e., age, self-reported sex [1 = Female], and race [1 = White]) were included in step 1. In step 2, depression and anxiety were included to evaluate their impact on grit while controlling for age, self-reported sex, and race. Resilience and hope were added in step 3 to determine their unique effects on grit, over and above depression and anxiety when controlling for the demographic variables.

## Results

### Descriptive Statistics

Descriptive statistics of participant's demographic and MS characteristics are presented in Table 1. Participants ranged in age from 21 to 77 years old with an average of 49.00 years old (*SD* = 11.63). Most participants identified as female (81.3%), Caucasian (92.5%), not of Hispanic, Latino, or Spanish origin (96.6%). More than half of the participants indicated they were married (62.6%), obtained a bachelor's, master's, or doctorate degree (63.8%), and were either full-time or part-time employed (52.6%). Regarding MS subtype, the majority of (77.3%) participants indicated having relapsing-remitting MS, followed by secondary progressive MS (11.2%), primary progressive MS (8.9%), and other (2.6%). Participants' average MS duration was 12.19 years (*SD* = 9.31).

**Table 1:** Participant demographic and MS characteristics (*N* = 348).

**Variable**	***n* (%)**	**Mean (SD)**
**Age**		49.00 (11.63)
**Self-reported sex**		
Female	283 (81.3%)	
Male	65 (18.7%)	
**Race**		
Caucasian	322 (92.5%)	
African American	11 (3.2%)	
Asian American/Pacific Islander	4 (1.1%)	
Native American/American Indian	1 (0.3%)	
Other	10 (2.9%)	
**Hispanic, Latino, Spanish Origin**		
Yes	12 (3.4%)	
No	336 (96.6%)	
**Marital status**		
Single	57 (16.4%)	
Married	218 (62.6%)	
Cohabitating	17 (4.9%)	
Widowed	6 (1.7%)	
Separated	4 (1.1%)	
Divorced	46 (13.2%)	
**Education level**		
Less than high school graduation	1 (0.3%)	
High school diploma or equivalency	29 (8.3%)	
Some college	61 (17.5%)	
Associate degree	35 (10.1%)	
Bachelor's degree	127 (36.5%)	
Master's degree	74 (21.3%)	
Doctorate degree	21 (6.0%)	
**Employment status**		
Full-time	160 (46.0%)	
Part-time	30 (8.6%)	
Unemployed	43 (12.4%)	
Retired	68 (19.5%)	
Student	3 (0.9%)	
Looking for work	5 (1.4%)	
Other	39 (11.2%)	
**MS subtype**		
Relapsing-remitting	269 (77.3%)	
Primary progressive	31 (8.9%)	
Secondary progressive	39 (11.2%)	
Other	9 (2.6%)	
**MS duration (years)**		12.19 (9.31)

Participants' mean depression score was 1.97 (*SD* = 1.78), and mean anxiety score was 2.76 (*SD* = 1.91). Participants' mean resilience score was 3.20 (*SD* = 0.81), and mean hope scores was 2.98 (*SD* = 0.49). Participants' mean grit score was 3.36 (*SD* =0 .59).

### Correlation Analysis

Grit was positively associated with resilience (*r* = 0.53, *p* < 0.001) and hope (*r* = 0.49, *p* < 0.001).

### Hierarchical Regression Analysis

Hierarchical regression analysis results are presented in Table 2. In step 1, age, self-reported sex, and race were entered. The amount of variance in grit scores accounted by the demographic variables was not significant: *R* = 0.15*, R*^2^ = 0.02, *F*(3, 344 = 2.19), *p* = *n.s*.

**Table 2:** Hierarchical regression analysis for prediction of grit (N = 348).

**Variable**	***R^**2**^***	***ΔR^**2**^***	***F***	**At entry into model**	**Final model**
				**β**	**β**
Step 1 (demographic)	0.02^+^	0.02	2.19		
Age				0.13^*^	0.06^+^
Self-reported sex^a^				−0.02^+^	0.03^+^
Race^b^				−0.02^+^	0.02^+^
Step 2 (risk factors)	0.21^***^	0.20	18.61		
Depression				−0.26^***^	−0.13^*^
Anxiety				−0.24^***^	−0.02^+^
Step 3 (protective factors)	0.33^***^	0.12	23.76		
Resilience				0.29^***^	0.29^***^
Hope				0.21^***^	0.22^**^

*** *p < 0.001*;

** *p < 0.01*;

* *p < 0.05*;

+ *p = n.s*.

a *Dummy coded self-reported sex (female = 1)*,

b *dummy coded race (White = 1)*.

In step 2, depression and anxiety were added. Depression and anxiety accounted for a significant amount of variance in grit even after controlling for age, self-reported sex, and race: *R* = 0.46*, R*^2^= 0.21, Δ*R*^2^ = 0.20, *F*(5, 342 = 18.61), *p* < 0.001. Furthermore, depression (β = −0.26, *p* < 0.001) and anxiety (β = −0.24, *p* < 0.001) were associated with grit, indicating lower depression and anxiety scores were associated with higher grit scores.

In step 3, resilience and hope were added. Resilience and hope accounted for a significant amount of variance beyond that explained by the demographic variables, depression, and anxiety: *R* = 0.57*, R*^2^= 0.33, Δ*R*^2^ = 0.12, *F*(7, 340 = 23.76), *p* < 0.001. Results demonstrated that resilience and hope explained 33% of variance in grit. Furthermore, the standardized beta coefficient for depression was reduced to −0.13, *p* < 0.05, and anxiety was reduced to −0.02, *p* = *n.s*. Resilience (β = 0.29, *p* < 0.001) and hope (β = 0.22, *p* < 0.01) were associated with grit, indicating higher resilience and hope scores were associated with higher grit scores.

## Discussion

The current study provided preliminary results that resilience and hope explained a significant amount of variance in grit when controlling for demographic covariates, depression, and anxiety. Consistent with previous research, resilience and hope were positively associated with grit (26, 40, 42). This study suggests that if people with MS have hope and resilience, their perseverance and passion to pursue long-term goals may increase despite adversity. As previous research has suggested, higher grit was associated with better health care management skills, mental and physical health quality of life, and lower depression and anxiety in people with chronic health conditions (25, 50). Those with higher grit may have better coping mechanisms and social support, and less impacted by stressors, contributing to better health outcomes (50). This is relevant to the present study in the sense that people with MS who have higher grit may have enhanced coping resources and skills, which can ultimately result in better health, mental health, and rehabilitation outcomes.

People with MS face multi-faceted challenges, including unpredictable symptoms, treatment complications, social relationships, employment, and financial stressors, which involves (51), which may involve people's resilience to face these obstacles and cope with them. Resilience has been found to negatively relate to depression, and positively relate to social and physical functioning and positive affect among individuals with MS (29, 43). This study demonstrated the positive association between resilience and grit, indicating that people with MS who had higher resilience were more likely to endorse higher grit. Resilience in people with disabilities has been described as their personal qualities to thrive and bounce back from adversity (52). People with MS can work on building resilience, such as utilizing personal and social skills and peer support functions (53). Bolstering resilience in people with MS may in turn lead to higher grit.

The shift in role changes can influence hope in people with MS as they might hope to be restored to their identity prior to MS (54). Research has also found hope was negatively linked to depression and anxiety, and was positively related to positive affect and life satisfaction in individuals with MS (33, 46). Finding of this study revealed the positive relationship between hope and grit, which suggests that people with MS who had higher levels of hope were more likely to exhibit higher levels of grit. Hope has been discussed as a coping resource where individuals with disabilities work toward improving their health conditions and wellbeing through goal-directed and self-determined motivational processes (52). Building hope might include seeking a life direction and reconstructing a sense of purpose, which people with MS can establish hope through embracing and accepting their value changes associated with MS (54). Through building hope in people with MS, this might in turn lead to increased levels of grit.

### Implications for Practice

Findings of the study provide implications for practice to assess and boost resilience, hope, and grit in individuals with MS. By gaining a better understanding of people with MS resilience, hope, and grit, rehabilitation professionals can better support their goals and psychosocial adjustment based on personal strengths and qualities. As resilience and hope have been identified as protective factors, rehabilitation professionals can assess resilience and hope in people with MS, which may help prevent the development of further negative outcomes. For instance, rehabilitation professionals can use resilience measures (e.g., *Brief Resilience Scale*) to directly assess resilience or assess resilience more holistically through identifying various factors that are indicative of resilience (55). Rehabilitation and mental health professionals (e.g., psychiatrists, psychologists, and rehabilitation counselors) can use Snyder's hope theory to understand and facilitate client's goals, strengths, and distress, and use the *Trait Hope Scale* to assess hope (56). Additionally, assessing grit may be beneficial in the identification of the potential negative consequences and determination of effective treatments (25). The assessment of resilience, hope, and grit can guide rehabilitation professionals in providing more suitable and individualized treatments that are most aligned with individuals with MS' desires, needs, and goals.

Resilience, hope, and grit are modifiable; therefore, rehabilitation professionals can integrate strength-based interventions into their practices to bolster resilience, hope, and grit in people with MS. Resilience interventions have shown promising results in improving resilience and mental health outcomes in people with MS (35, 36). Hope-based intervention has also led to increased hope and meaning in life, and reduced anxiety and depression (37). Although interventions to promote grit have predominantly taken place in academic settings (57, 58), researchers have discussed cognitive-behavioral therapy or value-based interventions may improve grit by helping individuals work on their thoughts and behaviors that are consistent with their long-term goals (25, 59). When working with individuals with MS, rehabilitation professionals can include those interventions to cultivate resilience, hope, and grit, which may potentially lead to enhanced health, mental health, and psychosocial outcomes.

Given recent research in psychiatry (60), psychology (61), and rehabilitation (45, 62, 63) have emphasized on positive protective factors, we believe that our findings will significantly benefit clinicians and researchers in mental health and rehabilitation. Jeste et al. (60) reported that as a new phenomenon in psychiatry, positive psychology/psychiatry research should be expanded in the context of mental health. They also emphasized that understanding and strengthening positive protective factors through interdisciplinary approaches (e.g., behavioral, social, biological interventions) has a great potential to improve mental health outcomes. Our study, therefore, contributes to the knowledge on positive psychology factors and their impact on people with disabilities.

### Limitations

There are several limitations to be considered in this study when interpreting results. A convenience sample of people with MS participated in the online survey recruited from the NMSS. Participants who had Internet access, were able to navigate an online survey, and were engaged with the NMSS, may be characteristically different from the broader MS population. Additionally, participants were predominantly White and well-educated in this study. For these reasons, this sample may not represent the general population of Americans with MS, which may limit the generalizability of the study's findings. The study also relied on self-report questionnaires to assess depression, anxiety, resilience, hope, and grit, which can be more susceptible to response bias and social desirability bias. To our knowledge, this was also the first study examining grit in people with MS, which future research would be warranted to further investigate grit and other outcomes (e.g., goals) in this population. Future studies may also consider comparing hope, resilience, and grit between people with MS and healthy controls. Since this study focused on resilience, hope, and grit, future research may consider adding other positive psychological variables. This was a cross-sectional study, causal relationships could not be inferred. Future longitudinal research design should further examine the relationships among resilience, hope, and grit.

## Conclusion

This preliminary study provides support for the relationships among resilience, hope, and grit in a sample of persons with MS. Findings suggested that resilience and hope were associated with grit when controlling for demographic variables, depression, and anxiety in a sample of people with MS. Moreover, implementing strength-based interventions may help enhance resilience, hope, and grit in people with MS, resulting in improved health, mental health, and psychosocial outcomes.

## Data Availability Statement

The original contributions presented in the study are included in the article/supplementary material, further inquiries can be directed to the corresponding author.

## Ethics Statement

The studies involving human participants were reviewed and approved by the University of Wisconsin-Madison. The patients/participants provided their written informed consent to participate in this study.

## Author Contributions

All authors listed have made a substantial, direct, and intellectual contribution to the work and approved it for publication.

## Funding

Support for this research was provided by the University of Wisconsin–Madison, Office of the Vice Chancellor for Research and Graduate Education with funding from the Wisconsin Alumni Research Foundation.

## Conflict of Interest

The authors declare that the research was conducted in the absence of any commercial or financial relationships that could be construed as a potential conflict of interest.

## Publisher's Note

All claims expressed in this article are solely those of the authors and do not necessarily represent those of their affiliated organizations, or those of the publisher, the editors and the reviewers. Any product that may be evaluated in this article, or claim that may be made by its manufacturer, is not guaranteed or endorsed by the publisher.
